# Soil Tillage Management Affects Maize Grain Yield by Regulating Spatial Distribution Coordination of Roots, Soil Moisture and Nitrogen Status

**DOI:** 10.1371/journal.pone.0129231

**Published:** 2015-06-22

**Authors:** Xinbing Wang, Baoyuan Zhou, Xuefang Sun, Yang Yue, Wei Ma, Ming Zhao

**Affiliations:** 1 Institute of Crop Sciences, Chinese Academy of Agricultural Sciences, Beijing, China; 2 College of Agronomy and Biotechnology, China Agricultural University, Beijing, China; Institute of Genetics and Developmental Biology, Chinese Academy of Sciences, CHINA

## Abstract

The spatial distribution of the root system through the soil profile has an impact on moisture and nutrient uptake by plants, affecting growth and productivity. The spatial distribution of the roots, soil moisture, and fertility are affected by tillage practices. The combination of high soil density and the presence of a soil plow pan typically impede the growth of maize (*Zea mays* L.).We investigated the spatial distribution coordination of the root system, soil moisture, and N status in response to different soil tillage treatments (NT: no-tillage, RT: rotary-tillage, SS: subsoiling) and the subsequent impact on maize yield, and identify yield-increasing mechanisms and optimal soil tillage management practices. Field experiments were conducted on the Huang-Huai-Hai plain in China during 2011 and 2012. The SS and RT treatments significantly reduced soil bulk density in the top 0–20 cm layer of the soil profile, while SS significantly decreased soil bulk density in the 20–30 cm layer. Soil moisture in the 20–50 cm profile layer was significantly higher for the SS treatment compared to the RT and NT treatment. In the 0-20 cm topsoil layer, the NT treatment had higher soil moisture than the SS and RT treatments. Root length density of the SS treatment was significantly greater than density of the RT and NT treatments, as soil depth increased. Soil moisture was reduced in the soil profile where root concentration was high. SS had greater soil moisture depletion and a more concentration root system than RT and NT in deep soil. Our results suggest that the SS treatment improved the spatial distribution of root density, soil moisture and N states, thereby promoting the absorption of soil moisture and reducing N leaching via the root system in the 20–50 cm layer of the profile. Within the context of the SS treatment, a root architecture densely distributed deep into the soil profile, played a pivotal role in plants’ ability to access nutrients and water. An optimal combination of deeper deployment of roots and resource (water and N) availability was realized where the soil was prone to leaching. The correlation between the depletion of resources and distribution of patchy roots endorsed the SS tillage practice. It resulted in significantly greater post-silking biomass and grain yield compared to the RT and NT treatments, for summer maize on the Huang-Huai-Hai plain.

## Introduction

The morphological conformation of the root system is an important contributor to crop productivity. It determines the ability of crops to explore and absorb the dynamic resources in the soil profile [1–3]. Competition for soil space and absorption of nutrients and moisture by roots is determined by the spatial distribution of roots [4]. Root morphology and spatial distribution are significantly affected by soil properties [5–6]. The spatial distribution of soil moisture and nutrients is affected by soil properties and the spatial distribution of roots [7]. High soil compaction has recently been identified as the main limiting factor for plant root growth and uptake of moisture and N [8–9].

In 2008, 916 representative soil samples were collected from 31 experimental stations in the three major corn-producing areas of 17 China provinces. The average soil bulk density was 1.39 g cm^-3^ at the 5–10 cm depth. In the soil plow pan, bulk density was 1.52 g cm^-3^. The average soil bulk density was much higher than the optimal soil bulk density, which is 1.1–1.3 g cm^-3^ for maize [10]. Soil with high bulk density and a plow pan is detrimental to maize root growth. High soil bulk density reduces soil porosity and uniformity of the soil structure [11,12]. Soil tillage and mechanical compaction result in variable soil properties throughout the soil profile [13], in particular the formation of a hard plow pan at a depth of about 15 cm [14]. Francois et al. [15] studied the influence of soil tillage management on spatial changes in soil physical and chemical properties. They reported that the plow pan disrupted the continuity of voids in the soil profile, resulting in reduced moisture content in the deep soil and large differences in moisture content throughout the soil profile layers.

Among the physical and chemical properties of soil, bulk density significantly influences root growth and extension [16,17]. Compacted soil with high bulk density reduces the rate and extent of root growth [18–20], shortens the length of roots in lower layers of the profile, enhances the length of roots in upper layers [21], and significantly impacts the spatial distribution of roots [22]. Differences in soil compaction among different layers could influence the vertical distribution of roots, and the influence on root length is greater than on dry weight [23]. The hard plow pan hinders root growth and extension [16,17], thereby limiting the distribution of roots and reducing absorption of moisture and nutrients from the deep soil layers. Soil compaction significantly influences absorption of soil moisture by the root system. The root absorption rate in moderately compacted soil is 67% higher than in highly compacted soil [23]. When soil moisture content decreases, high compaction impedes moisture absorption in the deep soil layers, limiting root growth, and similarly affecting crop growth [24].

A wide and deep distribution of the maize root system is required for optimal yield [25,26]. In a recent study, maize roots showed horizontal contraction and vertical extension, and the root systems in the deep soil layers significantly increased [27]. Wiesler and Horst [28] showed that the ability of maize to use moisture and nutrients in the deep soil is positively correlated with root length and density. Increased root growth in the deep layers enhances absorption of moisture and N [29]. The root architecture that confers efficient N uptake in maize is extensive and deep in the soil profile, it could have strong mediation ability and a large proportion of grain distribution [30,31]. Deep soil is relatively stable, and extension of the maize root system to this layer is conducive to increased root vitality and continued growth, contributing to the moisture and nutrient supply in the upper soil layers [32]. Deep roots could reduce N loss and enhance drought tolerance [33,34].

Available soil resources (moisture, N, and oxygen) also significantly influence root growth and distribution [22]. The response of roots to the spatial and temporal variation in soil moisture and nutrients also influences root growth and distribution [29,35,36]. Reductions in soil resources and increases in soil bulk density significantly influence root growth and distribution, and reduce moisture and nutrient absorption by roots [22].

Soil moisture and nutrient availability affect root growth, which has a significant impact on the spatial distribution of root systems [4]. Root mass is significantly greater in wet soil than in dry soil [37–39]. However, significant impediments to root depth, and the associated reduction in available soil moisture and fertility, contribute to increased lodging, premature aging, and reduced grain yield. Therefore, the coordination of root system distribution, and available soil moisture and N in the soil profile, are required for optimal plant growth and production [40].

In the present study, we compared the effects of different tillage management practices on the spatial distribution of root systems, soil moisture, and N status, and evaluated the effects of soil tillage management on the coordination of the root system and soil moisture spatial distribution. We also identified optimal tillage management practices to improve dry matter and grain yield through coordinating the spatial distribution of the root system and soil moisture/N status in the Huang-Huai-Hai region of China.

## Materials and Methods

### Site Description

The field experiments were conducted at the Xinxiang Experiment Station of the Chinese Academy of Agricultural Sciences, Henan Province, China (35°18’N, 113°54’E), in the 2011 and 2012 summer maize growing seasons. The experimental field was a typical double-cropping system in the Huang-Huai-Hai region. Winter wheat (*Triticum aestivum* L.) and summer maize are the main rotational crops in this area. The growing season of summer maize is from early June to late September. Prior to initiation of the experiment, the tillage systems were determined to be rotary-tillage (RT) for winter wheat and no-tillage (NT) for summer maize in the double-cropping system. The average temperature is 25.20°C during the summer growing season. Average annual precipitation is 567.3 mm, and more than 72% of the precipitation has occurred from June to September during the last 21 years. Soil texture is clay loam.

### Experimental Design and Field Management

The three soil tillage treatments in this field experiment were NT, RT, and SS. Prior to NT management, crop residues after wheat harvest were flattened and remained on the soil surface. The only disturbance to the soil was during planting and fertilizer application. The RT management treatment was done at a 10 cm depth after winter wheat harvest with a rotary tiller (1GKN-250, Yungangxuangengjixie Co. Ltd., Lianyungang, Jiangsu, China), and wheat residues were incorporated into the soil. The rotary tool bar was equipped with rotary blades spaced 75 cm apart, with a set of finishing disks behind the blade to break the large soil clods. The SS treatment involved complete soil inversion and burial of crop residue to a depth of 30 cm using a stripe deep loosening machine (Hehuinong machine Co. Ltd., Beijing, China). The experiment was conducted in two consecutive years at the same location. Initial soil water and nutrient contents, along the root zone profile for the three treatments, are shown in Table 1. Soil conditions at the beginning of the second season are provided in Table 2.

**Table 1:** Initial soil properties along the root zone profile before tillage treatments in 2011.

Soil properties	Soil depth (cm)				
	0–10	10–20	20–30	30–40	40–50
Bulk density (t/m^3^)	1.36	1.35	1.55	1.45	1.42
Soil moisture (g/g)	0.11	0.12	0.11	0.13	0.15
Organic C (g/kg)	13.37	11.73	5.95	6.53	6.21
Total N (g/kg)	1.19	1.07	0.83	0.75	0.69
Available P (mg/kg)	16.06	16.24	7.22	5.71	5.03
Available K (mg/kg)	119.78	100.12	93.65	107.26	98.37

SS, subsoiling; RT, rotary-tillage; NT, no-tillage.

**Table 2:** Soil conditions (soil bulk density and soil moisture)before the tillage treatment in 2012.

Treatment	Soil properties	Soil depth (cm)				
		0–10	10–20	20–30	30–40	40–50
SS	Bulk density (t/m^3^)	1.36	1.34	1.45	1.46	1.42
	Soil moisture (g/g)	0.11	0.11	0.12	0.12	0.16
RT	Bulk density (t/m^3^)	1.35	1.34	1.56	1.46	1.43
	Soil moisture (g/g)	0.09	0.11	0.12	0.12	0.16
NT	Bulk density (t/m^3^)	1.36	1.36	1.56	1.45	1.42
	Soil moisture (g/g)	0.11	0.12	0.11	0.12	0.16

SS, subsoiling; RT, rotary-tillage; NT, no-tillage.

The summer maize variety Zhengdan 958 was planted at a density of 45000 plants ha^-1^ with a 40–80 cm wide-narrow row spacing pattern. Seeds were sown by creating a 5 cm deep trench with a hand hoe for proper seed placement. After planting, the three treatments were given the same amount of irrigation to ensure similar initial soil water conditions. Nitrogen fertilizer was applied at 225 kg ha^-1^, and phosphate (P_2_O_5_) and potassium (K_2_O) fertilizers were applied at 75 and 150 kg ha^-1^, respectively. Among these fertilizers, 33% of the N fertilizer and all of the P_2_O_5_ and K_2_O fertilizers were broadcasted as basal fertilizers. The remaining N fertilizer was banded between the narrow rows as a top dressing at the jointing stage of maize. Irrigation was conducted twice in both 2011 and 2012. The first irrigation was at the jointing stage and the second application was 10 d after tassel emergence. Water was applied uniformly across treatments and years. With the exception of soil tillage, the remaining management practices were identical.

### Stalk sampling

Stalks were harvested at the growth stages of jointing (V6), tasseling (VT), and filling (R3) in 2011 and 2012. Grain was harvested from the middle six rows of each plot. From this sample, biomass was measured by drying the plants at 80°C to constant weight, and the yield components, including kernel numbers per ear and 1000-kernel weight, were recorded. The grain was dried at 80°C to record yield at 14% moisture content.

### Root sampling

**Fig 1:**
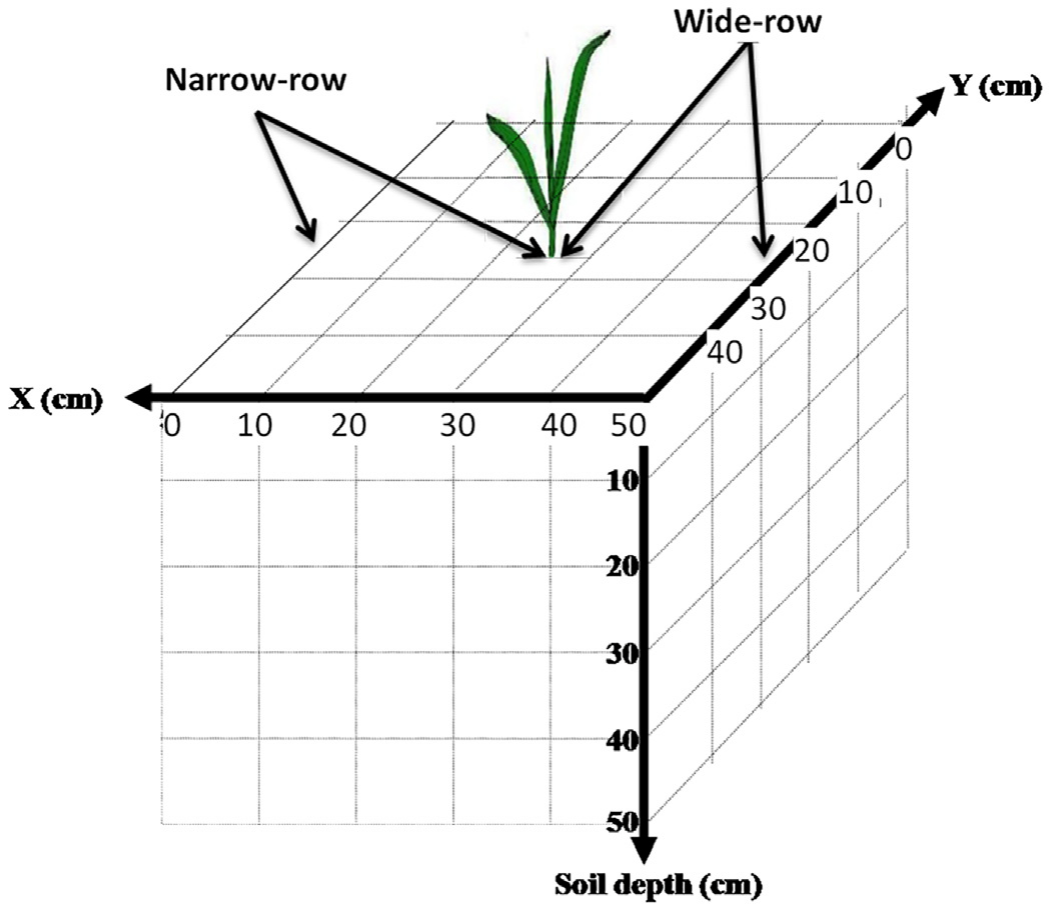
Diagrammatic representation of soil core sampling in the field plot.

After stalk sampling, root samples were taken according to a three-dimensional (3D) spatially distributed monolith scheme [41], which we then schematically depicted and illustrated with a photograph. Establishing the plant as the center of the sampled soil profile, the direction perpendicular to the planted row was designated the x-axis (0–25 cm in the narrow-row spacing, 25–50 cm in the wide-row spacing), the direction perpendicular to the planted row was the y-axis, and soil depth was the z-axis. The sampling unit was a block 10×10×10 cm in size. Soil volume was divided into 125 sub-volumes, each of which was 10×10×10 cm^3^ (Fig 1). All of the visible roots in each soil block were harvested by hand and put in individual labeled plastic bags. These roots were washed free of soil after transfer to the laboratory and then frozen at -5°C for subsequent analysis of root length. Root samples were harvested from different soil layers at each growth stage and scanned into images. The images were analyzed using the software WinRHIZO (Regent Instruments, 2009, Canada). After calculating root length, the root length density was recorded for each soil layer.

### Soil sampling

After removing the roots, the soil in each soil block was crushed by hand and sieved through a 3 mm sieve in the field. Soil moisture content was calculated as the difference between field-sampled and oven-dried (105°C for 24 h) soil, as follows: Soil moisture content (g g^-1^) = (soil fresh weight − soil dry weight) / soil fresh weight. Soil bulk density was measured according to the cutting-ring method and the following equation: Soil bulk density (g cm^-3^) = soil dry weight (g) / cutting-ring volume (cm^3^). Measurements were made at five soil depths (0–10, 10–20, 20–30, 30–40, and 40–50 cm) at the seedling stage. The measurements were repeated three times in each plot.

### Analytical methods

To study root length density, root dry-weight density, soil moisture content, and soil N content, the main focus was soil management at three levels, distance from the emitter at three levels, and depth at five levels. Analysis of variance was performed according to the general linear model (GLM) of SPSS v. 11.0 (SPSS Inc., 1996). Means were compared using the least significant difference (LSD) test at a probability level of 0.05.

Graphics on the spatial and temporal distribution of root length density, root dry-weight density, soil moisture content, and soil N content were obtained with Surfer10.0 software (Golden, CO, USA). The root architecture was simulated using the functional-structural plant model [42].

## Results

### Climate conditions in consecutive years

**Fig 2:**
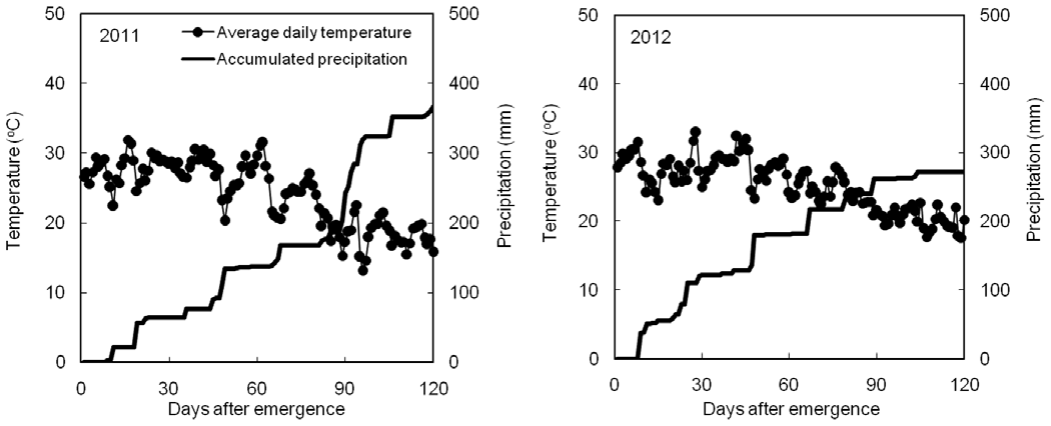
Changes of average temperature and accumulated precipitation during the growth stage of maize in 2011 and 2012.

Daily temperature and accumulated precipitation data, from emergence to harvest in 2011 and 2012, are shown in Fig 2. The accumulated precipitation during the growing seasons was 352.72 mm in 2011 and 272.83 mm in 2012. Growing season precipitation represented 61% and 76% of the total precipitation in 2011 and 2012, respectively, which met the water needs of the maize. The maximum temperature was from June to August in both years. The mean temperature during the growing season was 25.15°C and 25.90°C in 2011 and 2012, respectively.

### Grain yield and dry matter under different managements

Grain number, kernel weight, final grain yield, and grain N uptake were highest for the SS treatment, while there were no significant differences in the number of ears per hectare among the three soil tillage treatments (Table 3). Compared to RT and NT, the SS treatment increased grain yield by 6.89% and 8.56%, respectively, in 2011, and 10.83% and 12.46% in 2012. Correspondingly, compared to RT and NT, the SS treatment increased grain number by 5.43% and 4.79% in 2011, and 3.19% and 2.34%, in 2012. For 1000-kernel weight, the SS treatment was superior to RT and NT by 1.83% and 1.51% in 2011, and 9.62% and 8.50% in 2012. Compared to RT and NT, the SS treatment increased grain N uptake by 10.06% and 10.95% in 2011, and 14.19% and 14.57% in 2012.

**Table 3:** Effects of tillage managements on maize yield in two years.

Year	Tillage	Ear number(10^4^ hm^-2^)	Grain number per ear	1000-kernel weight (g)	Yield (t hm^-2^)	Nitrogen uptake (kg hm^-2^)
2011	SS	5.68a	579a	308.14a	9.77a	135.80a
	RT	5.53a	549b	302.60b	9.14b	123.39b
	NT	5.37a	553b	303.56b	9.00b	122.40b
2012	SS	5.30a	597a	362.22a	11.46a	155.87a
	RT	5.43a	556b	342.88b	10.34b	136.49b
	NT	5.22a	565b	345.71b	10.19b	136.04b

Different letters in the same column indicate significant differences at the 0.05 level (ANOVA and Duncan’s multiple range test), n = 3. SS, subsoiling; RT, rotary-tillage; NT, no-tillage.

The SS treatment significantly increased stalk and root dry matter, compared to RT and NT, in both years, while there was not a significant difference between the RT and NT treatments (Table 4). The greatest differences in stalk dry matter at the VT stage, when comparing the SS treatments to the RT and NT treatments, were 17.82% and 19.38% in 2011, and 18.20% and 20.89% in 2012. Root dry matter at V6 was greater for the SS treatment, compared to the RT and NT treatments, by 19.89% and 28.92% in 2011, and 25.38% and 21.68% in 2012.

**Table 4:** Effects of tillage managements on maize dry matter in two years.

Year	Tillage	Shoot dry matter (g)			Root dry matter (g)		
		V6	VT	R3	V6	VT	R3
2011	SS	73.42a	130.57a	337.95a	4.28a	16.82a	14.61a
	RT	64.27b	110.82b	308.85b	3.57b	14.34b	13.52b
	NT	63.53b	109.37b	305.43b	3.32b	14.41b	13.13b
2012	SS	83.78a	138.89a	326.67a	5.78a	17.19a	12.97a
	RT	75.99b	117.50b	286.75b	4.61b	15.68b	11.24b
	NT	73.11b	114.89b	279.56b	4.75b	14.75b	11.64b

Different letters in the same column indicate significant differences at the 0.05 level (ANOVA and Duncan’s multiple range test), n = 3. SS, subsoiling; RT, rotary-tillage; NT, no-tillage. V6, Jointing stage; VT, Tasseling stage; R3, Filling stage.

### Soil bulk density of different tillage treatments

**Fig 3:**
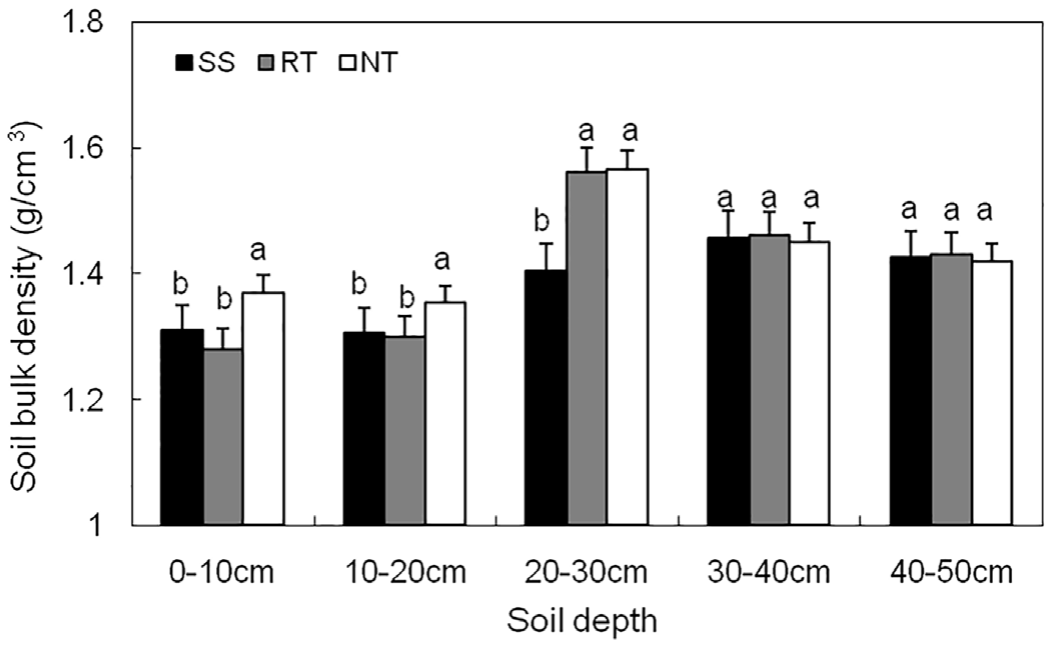
Soil bulk density in 0–50 cm depths at maize Tasseling stage under three tillage managements in 2012. Error bars represent as the standard error of the mean. Different letters above the error bars indicate significant differences at the 0.05 level (ANOVA and Duncan’s multiple range test), n = 3.

Soil bulk density was obviously modified by soil tillage (Fig 3 and Table A in S1 File). It was lower for the SS (0.05 g cm^-3^) and RT (0.07 g cm^-3^) treatments, compared to the NT treatment, in the 0–20 cm soil profile layer. Furthermore, the SS treatment significantly reduced soil bulk density, which was 0.16 g cm^-3^ greater than that of the RT and NT treatments in the 20–30 cm soil profile layer. There were no significant differences between the three tillage treatments in the 30–50 cm soil profile layer.

### Spatial distribution of soil moisture under different managements

**Fig 4:**
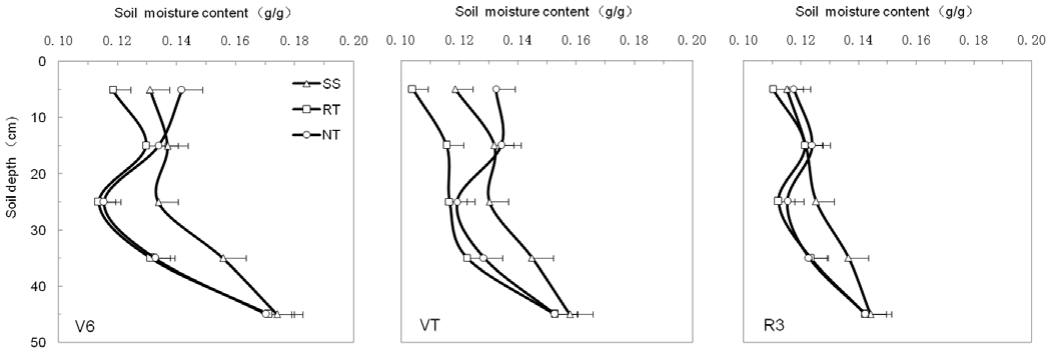
Soil moisture content(in the 50×50×10 cm^3^ soil volume) in 0–50 cm depths under three tillage managements in 2012. Error bars represent as the standard error of the mean.

Fig 4 (and Table B in S1 File) shows the soil moisture pattern in the 0–50 cm soil profile layer for the three tillage treatments at the V6, VT, and R3 maize growth stages. Generally, soil moisture was lowest in the 20–30 cm layer. The variation in soil moisture, as influenced by soil tillage, was not consistent in the 0–50 cm layer. In the 0–20 cm layer, NT had more moisture than the RT and SS treatments, while SS had more moisture than the RT and NT treatments in the 20–40 cm layer at all growth stages.

**Fig 5:**
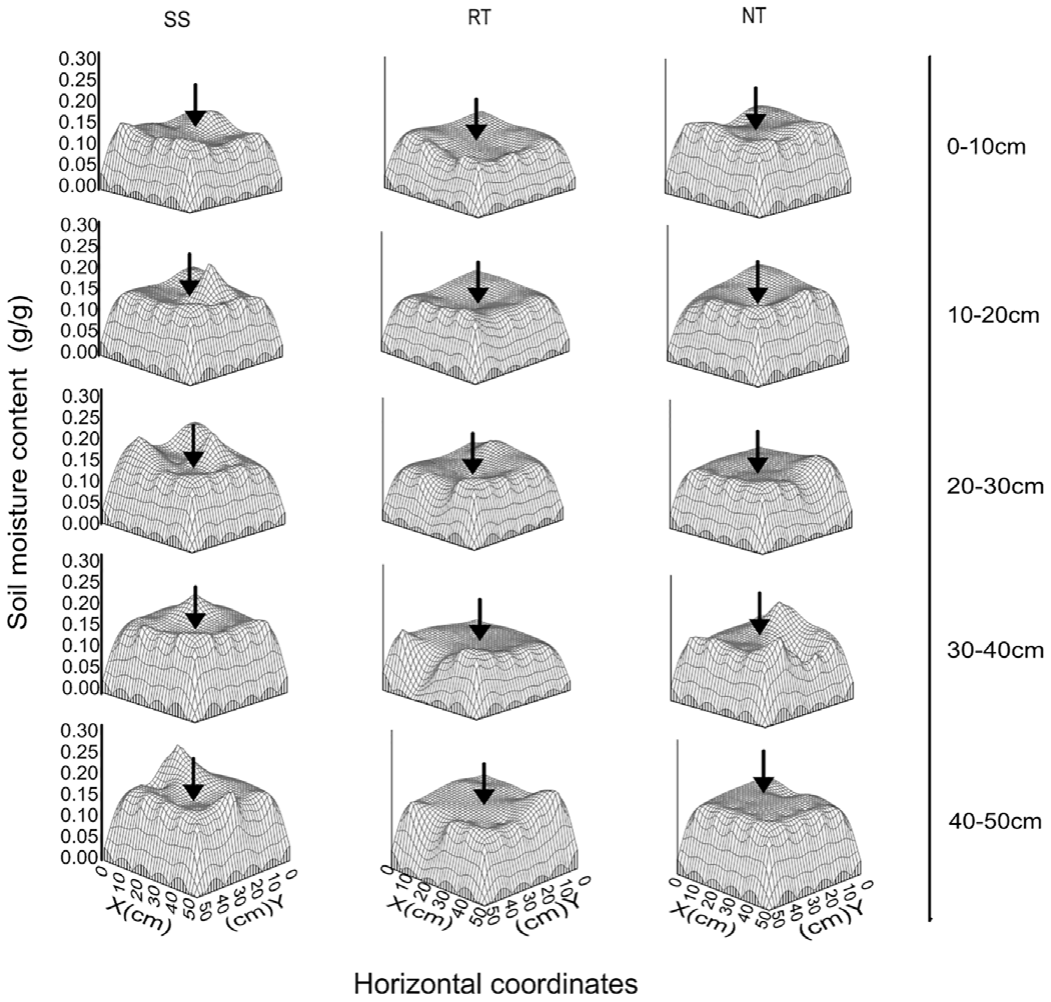
Spatial distribution of soil moisture in 0–50 cm depths at VT under three tillage managements in 2012. Solid arrows above each figure represent the position of maize growth centre.

The tillage management treatments had a strong and different influence on soil moisture in the 0–50 cm part of the soil profile at the VT growth stage (Fig 5 and Table C in S1 File). The RT treatment had the least soil moisture between the three tillage managements in the 0–50 cm layer. The SS treatment had more moisture (by 11.75%, 18.11%, and 3.47% respectively) in the 20–50 cm soil profile compared to NT. On the contrary, NT had more moisture (10.57%) in the 0–20 cm layer compared to the SS treatment (1.79%). Soil moisture content was less at the center of the plot, with greater moisture around the plot, especially in the 0–30 cm layer.

**Fig 6:**
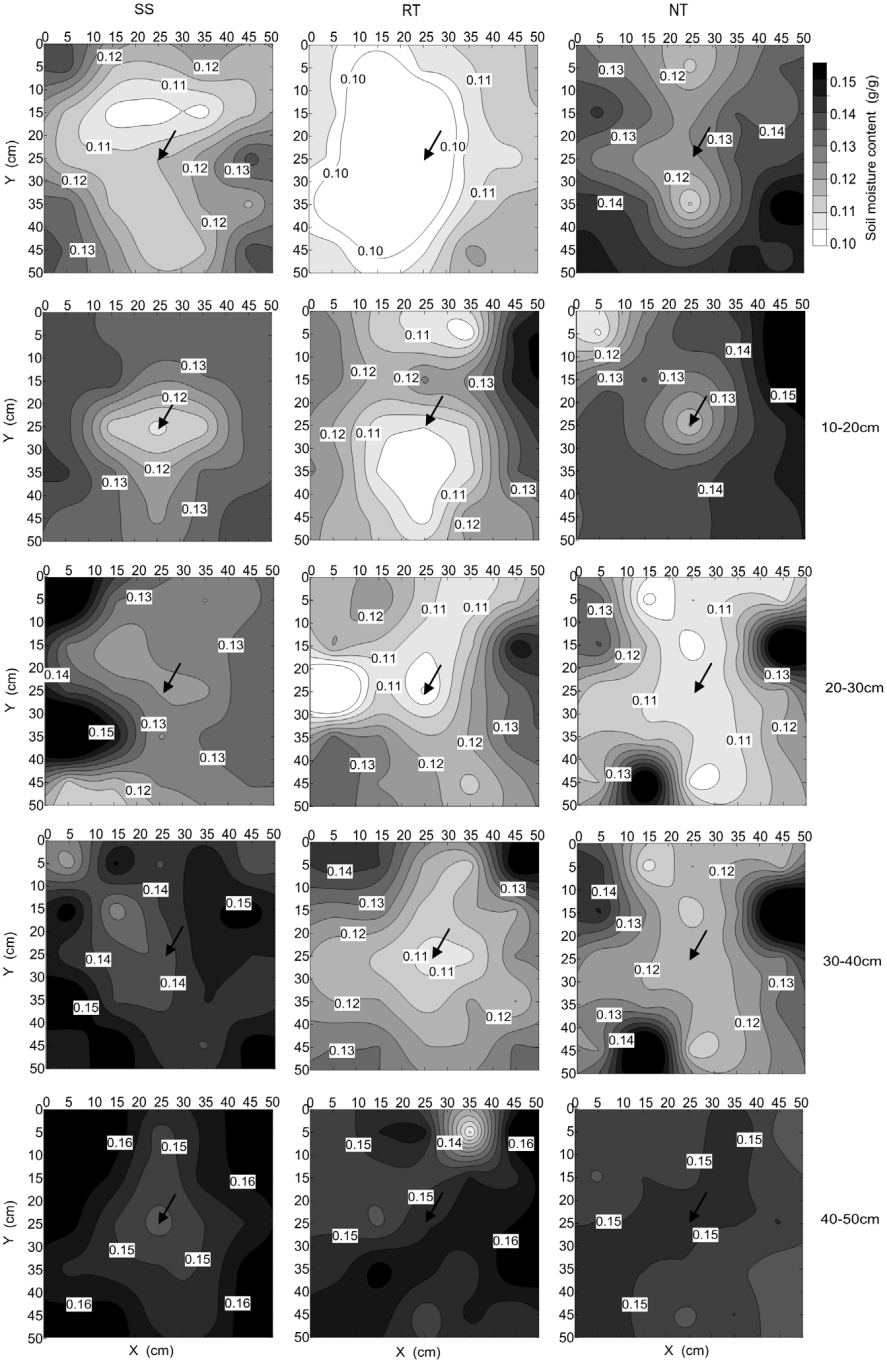
Horizontal distribution of soil moisture in each 10 cm soil profile at VT under three tillage managements in 2012. Solid arrows represent the position of maize growth centre.

Fig 6 presents the isogram distribution of soil moisture in each soil profile. Each profile across the 0–50 cm layer had low soil moisture in the center of the plot, which was identified as the moisture-depleted region. The spatial distribution of soil moisture under different tillage managements was variable in the 0–50 cm layer. For the SS treatment, the areas of depleted soil moisture were smaller than for the RT treatment, and throughout the soil profile. The SS treatment had reduced depletion areas in the 20–50 cm layer compared to the NT treatment. For the NT treatment, the depletion region was smaller than for the RT treatment in each layer. Compared to SS, only NT had less depletion in the 0–20 cm layer.

### Spatial distribution of the root system under different managements

**Fig 7:**
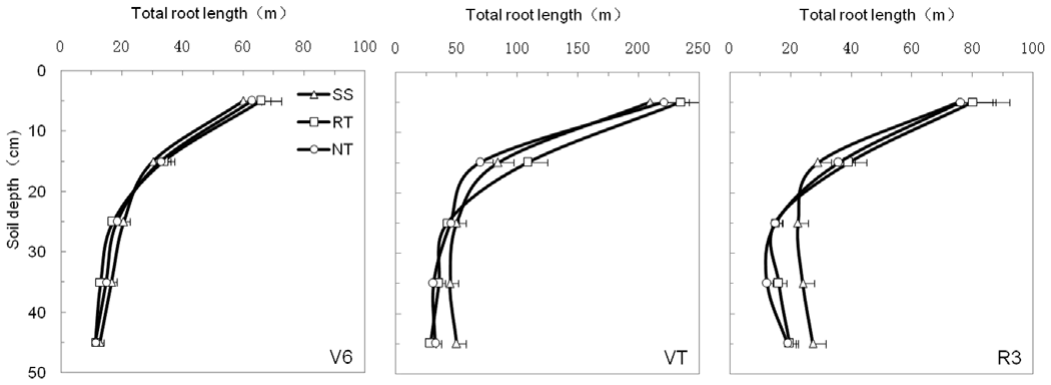
Total root length (in the 50×50×10 cm^3^ soil volume) in 0–50 cm depths under three tillage managements in 2012. Error bars represent as the standard error of the mean.

Fig 7 (and Table D in S1 File) shows the changes in root length in the 0–50 cm layer under each treatment for each growth stage. Generally, roots were longest in the 0–20 cm soil profile layer. The change in root length, as influenced by soil tillage, was not consistent in the 0–50 cm soil profile. In the 0–20 cm layer, the RT treatment led to longer roots than did the SS and NT treatments, while SS led to longer roots than the others in the 20–50 cm layer, especially at the VT and R3 maize growth stages.

**Fig 8:**
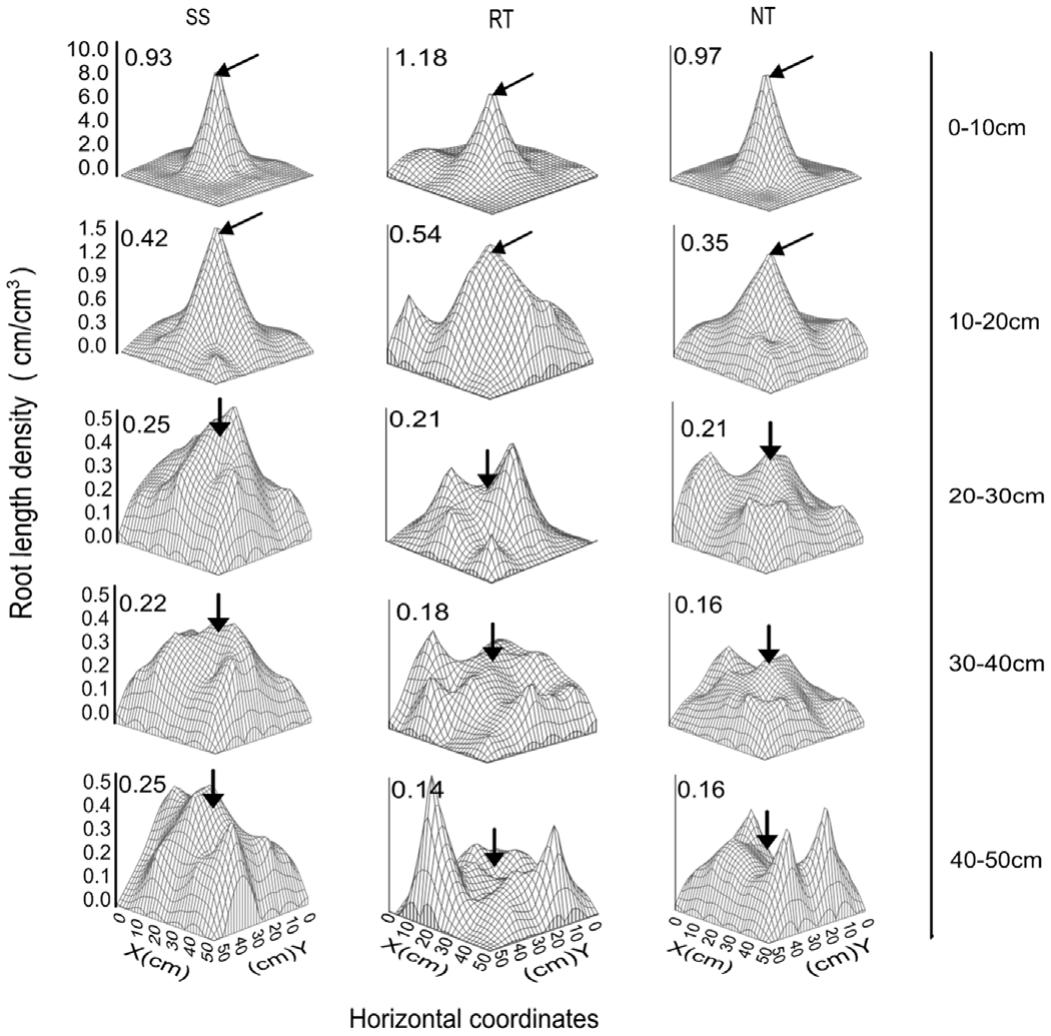
Spatial distribution of maize root in 0–50 cm depths at VT under three tillage managements in 2012. Numbers above each figure represent as the mean root length density in each soil depth. Solid arrows above each figure represent the position of maize growth centre.

The different tillage management practices had a marked influence on root length density in the 0–50 cm soil profile layer at the VT growth stage (Fig 8 and Table E in S1 File). The average root length density of SS and RT was 12.41% and 21.86% greater than that of the NT treatment, respectively. For the SS treatment, root length density was 18.57%, 26.58%, and 75.06% greater than that of RT for 20–50 cm soil layer, respectively, and 18.57%, 43.86%, and 53.12% greater than that of NT for 20–50 cm soil layer, respectively. The root length density in the 0–20 cm soil profile was 26.55% and 21.37% greater, and 28.90% and 56.48% greater, in RT than in SS and NT, respectively. In addition, NT management had the lowest root length density among the three treatments in the 10–50 cm layer. As shown in Fig 8, the distribution of root length in the 0–20 cm layer had a unimodal trend, with high root length density in the center of the plot and reduced density around the plot. In the 20–50 cm layer, the distribution of root length was multi-modal.

**Fig 9:**
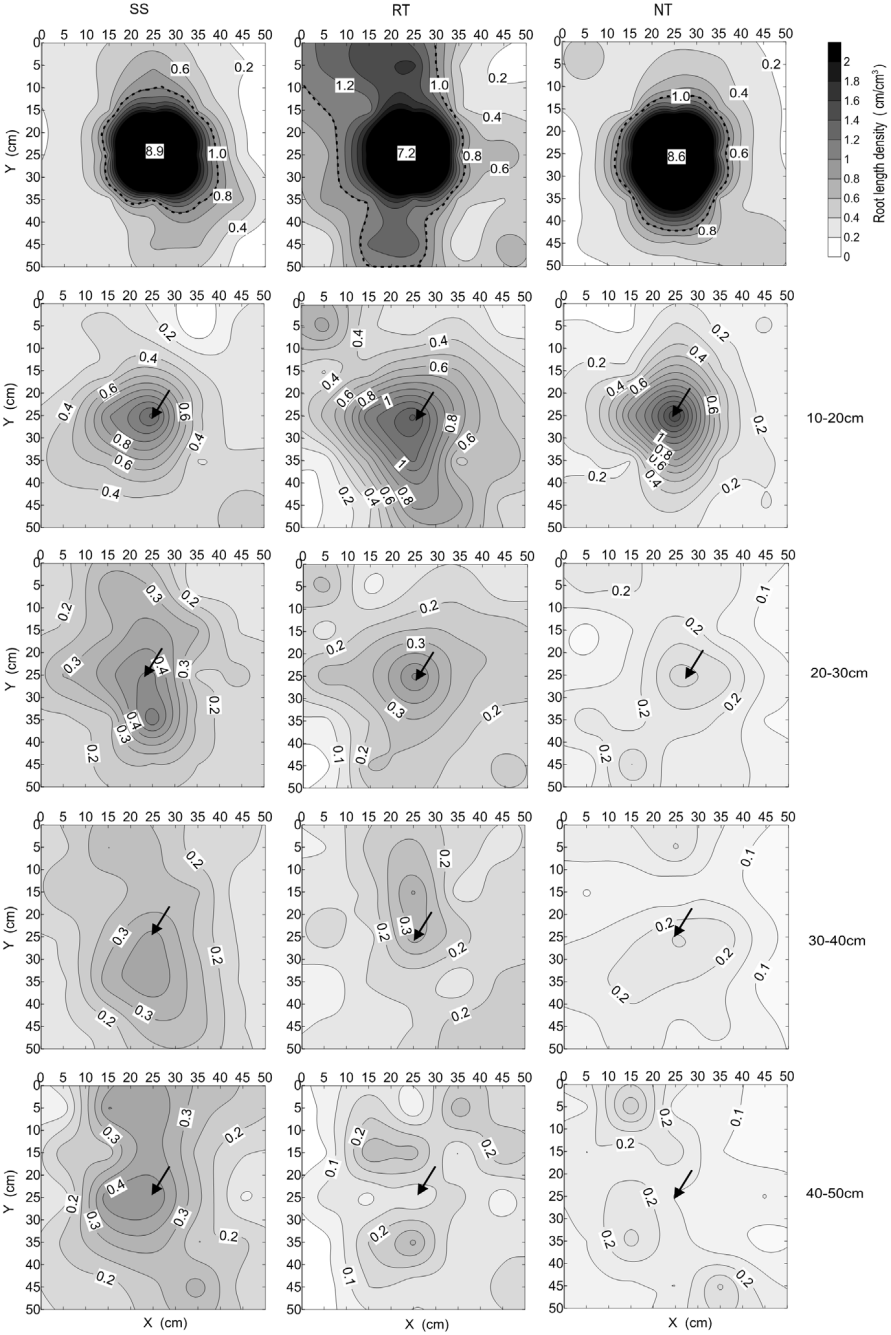
Horizontal distribution of maize root in 0–50 cm depths at VT under three tillage managements in 2012. Solid arrows above each figure represent the position of maize growth centre.

Fig 9 presents the isogram distribution of root length in each soil profile. Within the 0–50 cm profile, there was a high density of root length at the center of the plot, but the spatial distribution of root length varied throughout the layer depending on tillage treatment. Root length density was concentrated in the 20–50 cm layer for SS and in the 0–20 cm layer for RT. The density was less concentrated overall for NT management.

**Fig 10:**
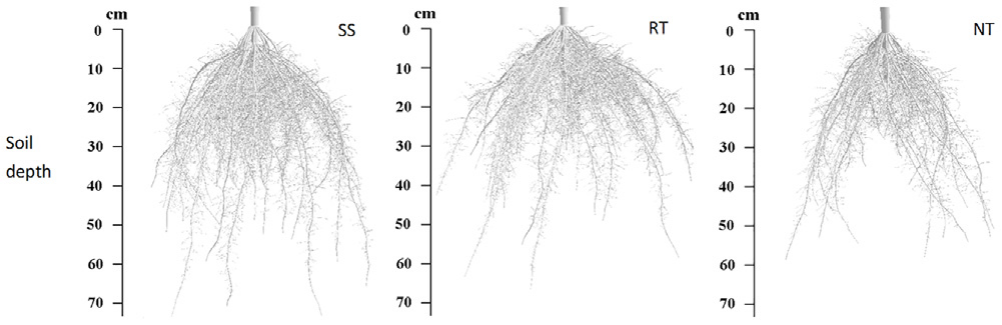
Simulated root architectures of maize at VT under three tillage managements in 2012.

The root architecture of maize, simulated using functional-structural plant models (FSPMs) at the VT stage under SS, RT, and NT management, are shown in Fig 10. There were differences in root spatial distribution and architecture for the different treatments. The root system was deeper for the SS treatment.

### Coordination distribution of soil moisture, total nitrogen, and maize root system

**Fig 11:**
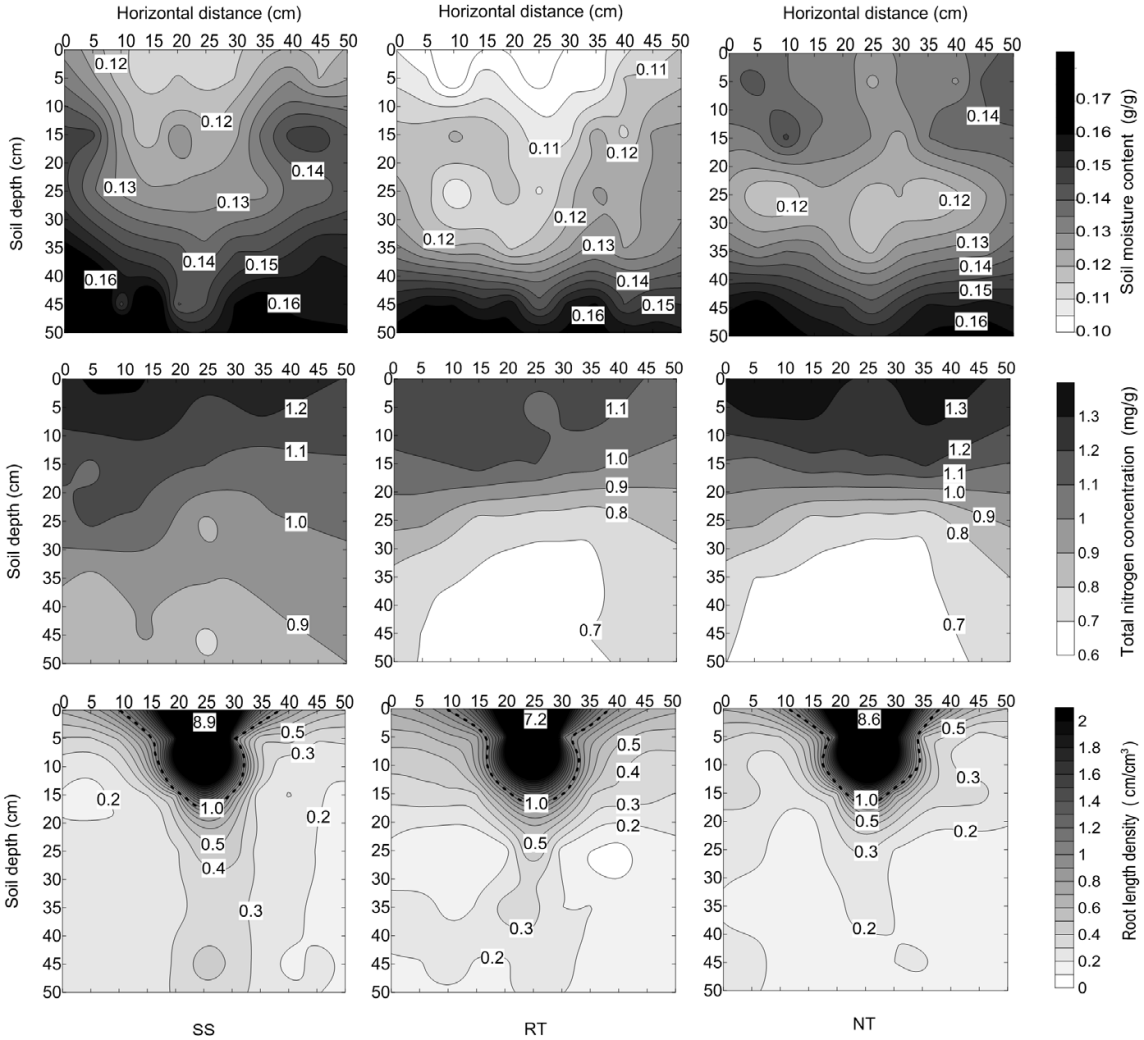
Coordination distribution of soil moisture (up), total nitrogen (middle) and root system (bottom) of maize in 0–50 cm depths at VT under three tillage managements in 2012.

The spatial distribution of soil moisture, total N, and the root system at 0–50 cm soil depths showed a different pattern (Fig 11 and Table F in S1 File). For all three tillage treatments, the soil moisture significantly increased from 10% to 16% while total N (1.4 to 0.6 mg g^-1^) and root length density (9 to 0.1 cm cm^-3^) significantly decreased with soil depth from 0 to 50 cm.

Soil moisture and total N concentration had the lowest values in the 0–50 cm soil depths under RT, while they showed the highest values in the 0–15 cm soil depths under NT and in the 15–50 cm soil depths under SS. In terms of vertical distribution, the root system was concentrated in the area depleted of moisture. In terms of the horizontal distribution, they were concentrated where both moisture and total N were depleted for all three soil tillage managements. In both the RT and NT treatments, the distributions of root systems were more horizontal than in the case of SS, following the same tendency as that observed for soil moisture content and total N concentration.

## Discussion

### Effects of soil tillage management on soil bulk density

Many studies have demonstrated that excessive soil tillage increases soil bulk density [43], and reduces soil moisture and nutrient availability [44,45]. He et al. [46] found that SS loosens the soil and prevents high soil bulk density. In the present study, SS significantly reduced soil bulk density in the top 0–30 cm layer of the soil profile, especially in the 20–30 cm layer, where it was superior to breaking the soil plow-pan, compared to the RT and NT treatments. This was because SS could eliminate soil compaction at the 0–20 cm depth and significantly break plow-pan layers at the 20–30 cm depth, while RT could only loosen the soil at the 0–20 cm depth.

### Effect of soil tillage management on distribution of soil moisture

Soil moisture infiltration can be improved by conservation tillage, with residue removal, compared to conventional tillage and NT [47]. Thierfelder and Wall [48] also found higher infiltration rates in conservation tillage than in conventional tillage. Excessive tillage practices move moist soil to the surface, leading to increased evaporation [49], whereas the reduced soil disturbance from conservation tillage decreases evaporation at the soil—atmosphere interface [50]. By increasing infiltration and reducing runoff and evaporation, compared to conventional tillage and NT, conservation agriculture can improve soil moisture and help buffer drought. In the present study, NT led to higher soil moisture than SS and RT in the 0–20 cm layer, while SS led to higher moisture levels than RT and NT in the 20–40 cm layer during all growth stages (Figs 4 and 5, and Tables B and C in S1 File). SS is a conservation tillage method that breaks the hard, high-bulk-density plow pan at the 20–30 cm layer, with little topsoil disturbance (Fig 3 and Table A in S1 File). As a result, the soil moisture can easily infiltrate from topsoil (0–20 cm layer) to subsoil (30–50 cm layer) through the plow pan (20–30 cm layer) in soil with high total porosity. Meanwhile, with little disturbance to the topsoil, the evaporation of surface soil moisture was very low due to mulching. The RT treatment was effective for flattening the surface soil in preparation for planting, with considerable disturbance and removal of mulch to the 0–20 cm layer. Topsoil with low soil bulk density and high topsoil porosity could easily be subject to high rates of evaporation in the absence of mulching. This lowers the moisture content in the 0–20 cm layer under RT, compared to the SS and NT treatments. NT had higher soil moisture content in the 0–20 cm layer because of reduced evaporation from the surface, with no topsoil disturbance or mulching. The NT treatment had lower moisture levels in the 20–40 cm layer compared to SS, due to the low moisture infiltration from topsoil to subsoil with high soil bulk density in the 20–30 cm layer.

Soil moisture had spatiotemporal heterogeneity, with high levels of spatial variability [51]. Spatial heterogeneity of soil moisture is an important hydraulic parameter that is also one of the most important factors for plant growth [52]. It depends on the soil’s capacity for infiltration, evaporation, and holding, governed by different environmental factors [53]. In general, moisture levels were lower nearer to plot and higher at 25 cm along the lateral from the plot, irrespective of tillage management (Fig 5 and Table C in S1 File). But the increment in moisture differed depending on soil tillage practice (Fig 6), showing a different horizontal distribution pattern under each practice. It was clear that the distribution of soil moisture was influenced by root absorption and moisture content, which led to moisture depletion in the 0–50 cm layer. In the 0–20 cm layer, RT had the greatest moisture depletion among the three tillage treatments. This could be attributed to the lower soil moisture content and more scattered root system distribution compared to the NT and SS treatments (Fig 7 and Table D in S1 File). On the other hand, although NT also had a scattered distribution of roots, it had the lowest moisture depletion area of the three tillage treatments. This was due to it leading to the highest levels of moisture in the 0–20 cm soil depth under NT compared to SS and RT. SS had moderate levels of moisture and a moderate root system distribution, and the area of depleted moisture was smaller than that of RT and larger than that of NT. In the 20–40 cm soil layer, SS had the smallest area of depleted moisture compared to RT and NT. Although the average values of root length density were highest under the SS treatment, the high soil moisture content led the small area of depleted moisture. The low soil moisture content, followed by absorption from the root system, resulted in a large area of depleted moisture under RT and NT. In the 40–50 cm soil layer, due to high moisture levels that could satisfy absorption by the root system, the soil profile had no apparent areas of depleted moisture for any treatment.

### Effects of soil tillage management on distribution of root system

In general, the increase in subsoil compaction resulted in a higher concentration of the root system in the upper soil layer, and reduced rooting in the deeper layers, due to the existence of a hard plow pan [54–56]. According to our results (Figs 7 and 10, and Table D in S1 File), because of the high soil bulk density in topsoil (0–20 cm layer) and a hard plow pan at the 20–30 cm soil depth, NT led to reduced root lengths in the 0–50 cm layer. For the RT treatment, although the hard plow pan limited root growth into deeper soil layers, the low soil bulk density promoted lateral root growth in the 0–20 soil layer. As a result, root length was greater in the 0–20 cm soil layer compared to the SS and NT treatments. SS is a conservation tillage method that can break the hard plow pan with little disturbance to topsoil (Fig 3 and Table A in S1 File). Consequently, roots can easily grow from topsoil (0–20 cm depth) to the subsoil (30–50 cm depth) through the plow pan (20–30 cm depth) under the SS treatment. This was the reason that SS led to extensive root lengths in the 20–50 cm layer.

The spatial distribution of root systems in a soil profile has a great influence on a crop’s capacity to absorb soil moisture and nutrients, and consequently, influences growth and productivity [57,58]. Soil tillage influences root growth and distribution [59]. Many previous studies have reported that the spatial distribution of maize roots is significantly influenced by soil tillage [60–62], which is the most important role in soil—plant systems. In the present study, the root system showed different horizontal distribution patterns for the three soil tillage treatments in different profile layers. Therefore, the area of root system “concentration region” was the obvious performance (Figs 8 and 9, and Table E in S1 File). In the 0–20 cm layer, RT had the largest area of root concentration among the tillage treatments. This can be attributed to greater root system growth compared to SS and NT (Fig 7 and Table D in S1 File). The scattered root system distribution would spread the area of the root system capable of absorbing soil moisture. On the contrary, due to restrictions in root system growth with high soil bulk density under NT, it had the smallest area of concentrated root growth. For SS, the small concentrated area of roots could have been due to more root growth in the deep soil layer, with steeper root growth angles. In the 20–50 cm layer, SS management led to the largest area of root concentration. The main reason is because this treatment breaks the hard plow pan, which otherwise limits the root system growth to the 20–30 cm soil layer. However, for RT and NT, only parts of the root systems could extend across the hard plow pan. This resulted in smaller areas of concentration root growth compared to SS.

### The distribution coordination of soil moisture, total N and the root system with different tillage managements

Tillage management practices have an impact on soil total N storage [63]. Grant [64] reported that conventional tillage hastens N mineralization. NT management has been shown to increase N concentration in soil profiles compared to the decrease after conventional tillage [65]. In the present study (Fig 11 and Table F in S1 File), SS had high values of total N concentration in the 20–50 cm soil layer. However, RT and NT had lower total N concentrations in the 20–50 cm layer compared to SS. On the other hand, due to the absence of mulching, soil N can be washed away via rainfall. Therefore, total N was lower under RT than for the SS and NT treatments.

The increment of soil compaction is a severe limiting factor altering moisture and nutrient distribution in the soil, thereby limiting root growth and crop productivity [66–68]. The spatial distribution of soil moisture, total N, and the root system had a coordinated relationship in the soil profile under SS (Fig 11 and Table F in S1 File). Because of the absorption of soil moisture and N by maize roots, the region where the root system was concentrated was depleted in spatial distribution of soil moisture and total N. In the 0–30 cm layer, dry soil was due to large absorption by the concentrated root system, while in the 30–50 cm layer, the region devoid of total N was due to absorption by the root system. Compared to RT and NT, SS management increased root length density, soil moisture content, and total N concentration in the 20–50 cm soil layer. Adequate soil moisture and N for root systems in the deep soil layer were very important at or after the tasseling growth stage. The spatial distribution of soil moisture, total N, and the root system of maize demonstrated a coordinated relationship. On the contrary, for RT and NT, the highest moisture content was in the 40–50 cm layer, but total N concentration and root length were the lowest in that layer. The spatially coordinated distribution of moisture, total N, and the maize root system was not sufficient in the RT and NT treatments.

### Effects of soil tillage management on maize dry matter and grain yield

Soil tillage practices significantly influence soil properties and root distribution [69,70], affecting crop dry matter and grain yield [71]. As shown in our field experiments, because of the lower soil bulk density, the growth of roots in the deep soil profile layers contributed to greater root dry matter and stalks under the SS treatment compared to the RT and NT treatments, particularly after the VT stage (Table 4). Moreover, SS had the greatest impact on grain yield and N content, with the highest increment of grain weight among the three tillage treatments (Table 3). Deeper and longer roots, and higher root length density and biomass, led to a greater uptake of deep soil moisture and absorption of soil N by the root system lower in the soil profile. Consequently, the coordinated distribution of moisture, total N, and the root system led to greater crop biomass and grain yield from SS, compared to RT and NT.

## Conclusions

The correlation between the depletion of resources and distribution of patchy roots revealed the strength of SS tillage. This research compared three tillage systems in the Huang-Huai-Hai plain, which had soil with a hard plow pan. SS helped to break the plow pan, accommodating soil moisture and nutrient access through increased root length in deep soil. SS tillage in the soil with a plow pan enhances soil moisture and nutrient uptake by promoting root growth in deep soil. There was also increased dry matter post-silking, resulting in improved soil environment conditions. In summary, this knowledge may contribute to breeding for deeper root traits, enabling more efficient acquisition of soil resources and synchronizing crop growth demand, root resource acquisition, and fertilizer application during the growing season, thereby maximizing crop yields and nutrient-use efficiency, and minimizing environmental pollution.

## Supporting Information

S1 File This file contains Tables A-F. **Table A**. Soil bulk density in 0–50 cm depths at VT under three tillage managements in 2011. **Table B**. Soil moisture content (in the 50×50×10 cm^3^ soil volume) in 0–50 cm depths under three tillage managements in 2011. **Table C**. Spatial distribution of soil moisture in 0–50 cm depths at VT under three tillage managements in 2011. **Table D**. Total root length (in the 50×50×10 cm^3^ soil volume) in 0–50 cm depths under three tillage managements in 2011. **Table E**. Spatial distribution of maize root in 0–50 cm depths at VT under three tillage managements in 2011. **Table F**. Coordination distribution of soil moisture, total nitrogen and root system of maize in 0–50 cm depths at VT under three tillage managements in 2011.
(DOCX)
