# Supplementary material for: Soil Tillage Management Affects Maize Grain Yield by Regulating Spatial Distribution Coordination of Roots, Soil Moisture and Nitrogen Status
Source: PLoS One. 2015 Jun 22;10(6):e0129231. doi: 10.1371/journal.pone.0129231 (PMC4476672; doi:10.1371/journal.pone.0129231)
Supplement: S1 File — Table A. Soil bulk density in 0–50 cm depths at VT under three tillage managements in 2011. Table B. Soil moisture content (in the 50×50×10 cm3 soil volume) in 0–50 cm depths under three tillage managements in 2011. Table C. Spatial distribution of soil moisture in 0–50 cm depths at VT under three tillage managements in 2011. Table D. Total root length (in the 50×50×10 cm3 soil volume) in 0–50 cm depths under three tillage managements in 2011. Table E. Spatial distribution of maize root in 0–50 cm depths at VT under three tillage managements in 2011. Table F. Coordination distribution of soil moisture, total nitrogen and root system of maize in 0–50 cm depths at VT under three tillage managements in 2011. (DOCX) [file pone.0129231.s001.docx]

| Soil depth (cm) | Soil bulk density (g/cm^3^) | | |
| --- | --- | --- | --- |
|  | SS | RT | NT |
| 0-10 | 1.32 | 1.3 | 1.36 |
| 10-20 | 1.31 | 1.31 | 1.35 |
| 20-30 | 1.42 | 1.56 | 1.55 |
| 30-40 | 1.46 | 1.45 | 1.45 |
| 40-50 | 1.42 | 1.43 | 1.42 |

**Table A.** Soil bulk density in 0–50 cm depths at VT under three tillage managements in 2011

**Table B.** Soil moisture content (in the 50×50×10 cm^3^ soil volume) in 0–50 cm depths under three tillage managements in 2011

| Plant growth stage | Soil depth (cm) | Soil moisture content (g/g) | | |
| --- | --- | --- | --- | --- |
|  |  | SS | RT | NT |
| V6 | 0-10 | 0.12 | 0.11 | 0.13 |
|  | 10-20 | 0.12 | 0.11 | 0.13 |
|  | 20-30 | 0.13 | 0.11 | 0.12 |
|  | 30-40 | 0.15 | 0.14 | 0.13 |
|  | 40-50 | 0.15 | 0.16 | 0.15 |
| VT | 0-10 | 0.10 | 0.11 | 0.12 |
|  | 10-20 | 0.12 | 0.11 | 0.12 |
|  | 20-30 | 0.12 | 0.12 | 0.11 |
|  | 30-40 | 0.14 | 0.12 | 0.13 |
|  | 40-50 | 0.14 | 0.14 | 0.15 |
| R3 | 0-10 | 0.19 | 0.18 | 0.17 |
|  | 10-20 | 0.18 | 0.18 | 0.18 |
|  | 20-30 | 0.18 | 0.16 | 0.15 |
|  | 30-40 | 0.15 | 0.15 | 0.14 |
|  | 40-50 | 0.16 | 0.16 | 0.14 |

**Table C.** Spatial distribution of soil moisture in 0–50 cm depths at VT under three tillage managements in 2011

| Soil moisture content (g/g) | | | | | | | |
| --- | --- | --- | --- | --- | --- | --- | --- |
| Soil tillage | Horizontal direction (cm) | | Soil depth (cm) | | | | |
|  | X | Y | 0-10 | 10-20 | 20-30 | 30-40 | 40-50 |
| SS | 0-10 | 0-10 | 0.10 | 0.13 | 0.12 | 0.14 | 0.14 |
|  | 0-10 | 10-20 | 0.11 | 0.13 | 0.12 | 0.14 | 0.14 |
|  | 0-10 | 20-30 | 0.11 | 0.12 | 0.13 | 0.16 | 0.13 |
|  | 0-10 | 30-40 | 0.11 | 0.12 | 0.13 | 0.14 | 0.15 |
|  | 0-10 | 40-50 | 0.11 | 0.12 | 0.13 | 0.14 | 0.14 |
|  | 10-20 | 0-10 | 0.11 | 0.13 | 0.13 | 0.13 | 0.17 |
|  | 10-20 | 10-20 | 0.10 | 0.14 | 0.13 | 0.14 | 0.15 |
|  | 10-20 | 20-30 | 0.10 | 0.12 | 0.11 | 0.14 | 0.18 |
|  | 10-20 | 30-40 | 0.11 | 0.12 | 0.12 | 0.15 | 0.12 |
|  | 10-20 | 40-50 | 0.10 | 0.13 | 0.13 | 0.14 | 0.14 |
|  | 20-30 | 0-10 | 0.11 | 0.13 | 0.11 | 0.14 | 0.15 |
|  | 20-30 | 10-20 | 0.09 | 0.13 | 0.12 | 0.15 | 0.14 |
|  | 20-30 | 20-30 | 0.11 | 0.11 | 0.13 | 0.14 | 0.13 |
|  | 20-30 | 30-40 | 0.10 | 0.11 | 0.11 | 0.14 | 0.13 |
|  | 20-30 | 40-50 | 0.10 | 0.12 | 0.14 | 0.14 | 0.14 |
|  | 30-40 | 0-10 | 0.11 | 0.14 | 0.12 | 0.13 | 0.16 |
|  | 30-40 | 10-20 | 0.11 | 0.13 | 0.12 | 0.13 | 0.13 |
|  | 30-40 | 20-30 | 0.10 | 0.12 | 0.13 | 0.14 | 0.13 |
|  | 30-40 | 30-40 | 0.09 | 0.12 | 0.11 | 0.14 | 0.13 |
|  | 30-40 | 40-50 | 0.11 | 0.12 | 0.12 | 0.13 | 0.14 |
|  | 40-50 | 0-10 | 0.11 | 0.13 | 0.11 | 0.12 | 0.13 |
|  | 40-50 | 10-20 | 0.12 | 0.12 | 0.12 | 0.12 | 0.13 |
|  | 40-50 | 20-30 | 0.11 | 0.11 | 0.11 | 0.13 | 0.18 |
|  | 40-50 | 30-40 | 0.11 | 0.11 | 0.12 | 0.15 | 0.14 |
|  | 40-50 | 40-50 | 0.13 | 0.11 | 0.12 | 0.15 | 0.15 |
| RT | 0-10 | 0-10 | 0.11 | 0.12 | 0.14 | 0.12 | 0.16 |
|  | 0-10 | 10-20 | 0.08 | 0.09 | 0.12 | 0.12 | 0.13 |
|  | 0-10 | 20-30 | 0.10 | 0.10 | 0.12 | 0.14 | 0.13 |
|  | 0-10 | 30-40 | 0.11 | 0.11 | 0.11 | 0.12 | 0.13 |
|  | 0-10 | 40-50 | 0.11 | 0.12 | 0.13 | 0.12 | 0.17 |
|  | 10-20 | 0-10 | 0.11 | 0.11 | 0.12 | 0.12 | 0.15 |
|  | 10-20 | 10-20 | 0.08 | 0.14 | 0.12 | 0.13 | 0.14 |
|  | 10-20 | 20-30 | 0.09 | 0.11 | 0.11 | 0.13 | 0.13 |
|  | 10-20 | 30-40 | 0.11 | 0.12 | 0.12 | 0.13 | 0.14 |
|  | 10-20 | 40-50 | 0.11 | 0.11 | 0.12 | 0.13 | 0.15 |
|  | 20-30 | 0-10 | 0.10 | 0.12 | 0.11 | 0.10 | 0.14 |
|  | 20-30 | 10-20 | 0.08 | 0.10 | 0.11 | 0.10 | 0.16 |
|  | 20-30 | 20-30 | 0.08 | 0.10 | 0.11 | 0.10 | 0.14 |
|  | 20-30 | 30-40 | 0.08 | 0.10 | 0.11 | 0.13 | 0.13 |
|  | 20-30 | 40-50 | 0.10 | 0.12 | 0.12 | 0.13 | 0.13 |
|  | 30-40 | 0-10 | 0.11 | 0.12 | 0.11 | 0.11 | 0.13 |
|  | 30-40 | 10-20 | 0.10 | 0.10 | 0.12 | 0.10 | 0.14 |
|  | 30-40 | 20-30 | 0.08 | 0.10 | 0.11 | 0.10 | 0.15 |
|  | 30-40 | 30-40 | 0.11 | 0.12 | 0.13 | 0.15 | 0.14 |
|  | 30-40 | 40-50 | 0.08 | 0.09 | 0.13 | 0.13 | 0.14 |
|  | 40-50 | 0-10 | 0.12 | 0.10 | 0.12 | 0.15 | 0.14 |
|  | 40-50 | 10-20 | 0.11 | 0.11 | 0.12 | 0.16 | 0.15 |
|  | 40-50 | 20-30 | 0.10 | 0.10 | 0.10 | 0.15 | 0.15 |
|  | 40-50 | 30-40 | 0.10 | 0.11 | 0.09 | 0.14 | 0.15 |
|  | 40-50 | 40-50 | 0.11 | 0.10 | 0.09 | 0.16 | 0.13 |
| NT | 0-10 | 0-10 | 0.12 | 0.12 | 0.11 | 0.12 | 0.16 |
|  | 0-10 | 10-20 | 0.12 | 0.12 | 0.12 | 0.13 | 0.15 |
|  | 0-10 | 20-30 | 0.10 | 0.13 | 0.11 | 0.12 | 0.15 |
|  | 0-10 | 30-40 | 0.12 | 0.12 | 0.14 | 0.15 | 0.14 |
|  | 0-10 | 40-50 | 0.13 | 0.11 | 0.13 | 0.14 | 0.15 |
|  | 10-20 | 0-10 | 0.13 | 0.13 | 0.17 | 0.18 | 0.15 |
|  | 10-20 | 10-20 | 0.12 | 0.12 | 0.11 | 0.12 | 0.15 |
|  | 10-20 | 20-30 | 0.11 | 0.12 | 0.11 | 0.12 | 0.15 |
|  | 10-20 | 30-40 | 0.13 | 0.12 | 0.12 | 0.13 | 0.15 |
|  | 10-20 | 40-50 | 0.12 | 0.12 | 0.10 | 0.11 | 0.15 |
|  | 20-30 | 0-10 | 0.12 | 0.12 | 0.10 | 0.11 | 0.14 |
|  | 20-30 | 10-20 | 0.11 | 0.12 | 0.11 | 0.12 | 0.15 |
|  | 20-30 | 20-30 | 0.11 | 0.10 | 0.11 | 0.12 | 0.15 |
|  | 20-30 | 30-40 | 0.13 | 0.11 | 0.10 | 0.11 | 0.15 |
|  | 20-30 | 40-50 | 0.13 | 0.13 | 0.11 | 0.12 | 0.15 |
|  | 30-40 | 0-10 | 0.13 | 0.13 | 0.11 | 0.12 | 0.15 |
|  | 30-40 | 10-20 | 0.13 | 0.12 | 0.11 | 0.12 | 0.15 |
|  | 30-40 | 20-30 | 0.11 | 0.12 | 0.11 | 0.12 | 0.15 |
|  | 30-40 | 30-40 | 0.12 | 0.12 | 0.11 | 0.12 | 0.15 |
|  | 30-40 | 40-50 | 0.15 | 0.12 | 0.11 | 0.12 | 0.15 |
|  | 40-50 | 0-10 | 0.14 | 0.13 | 0.12 | 0.13 | 0.14 |
|  | 40-50 | 10-20 | 0.13 | 0.13 | 0.12 | 0.13 | 0.14 |
|  | 40-50 | 20-30 | 0.13 | 0.13 | 0.12 | 0.13 | 0.15 |
|  | 40-50 | 30-40 | 0.14 | 0.14 | 0.18 | 0.19 | 0.15 |
|  | 40-50 | 40-50 | 0.13 | 0.15 | 0.11 | 0.12 | 0.14 |

| Plant growth stage | Soil depth (cm) | Total root length (m) | | |
| --- | --- | --- | --- | --- |
|  |  | SS | RT | NT |
| V6 | 0-10 | 65.92 | 72.80 | 70.74 |
|  | 10-20 | 36.40 | 41.71 | 39.97 |
|  | 20-30 | 26.33 | 22.10 | 22.50 |
|  | 30-40 | 20.25 | 17.79 | 15.96 |
|  | 40-50 | 15.83 | 14.37 | 14.42 |
| VT | 0-10 | 210.70 | 238.02 | 230.98 |
|  | 10-20 | 91.85 | 134.83 | 101.45 |
|  | 20-30 | 71.76 | 60.91 | 65.37 |
|  | 30-40 | 69.03 | 59.49 | 53.16 |
|  | 40-50 | 64.94 | 46.85 | 42.79 |
| R3 | 0-10 | 76.19 | 80.32 | 86.19 |
|  | 10-20 | 35.87 | 39.24 | 45.87 |
|  | 20-30 | 21.41 | 15.16 | 15.16 |
|  | 30-40 | 19.50 | 12.28 | 11.28 |
|  | 40-50 | 11.84 | 9.02 | 10.03 |

**Table D.** Total root length (in the 50×50×10 cm^3^ soil volume) in 0–50 cm depths under three tillage managements in 2011

**Table E.** Spatial distribution of maize root in 0–50 cm depths at VT under three tillage managements in 2011

| Root length density (cm/cm^3^) | | | | | | | |
| --- | --- | --- | --- | --- | --- | --- | --- |
| Soil tillage | Horizontal direction (cm) | | Soil depth (cm) | | | | |
|  | X | Y | 0-10 | 10-20 | 20-30 | 30-40 | 40-50 |
| SS | 0-10 | 0-10 | 0.07 | 0.04 | 0.14 | 0.15 | 0.20 |
|  | 0-10 | 10-20 | 0.18 | 0.45 | 0.25 | 0.23 | 0.12 |
|  | 0-10 | 20-30 | 0.10 | 0.38 | 0.17 | 0.40 | 0.60 |
|  | 0-10 | 30-40 | 0.47 | 0.16 | 0.20 | 0.18 | 0.39 |
|  | 0-10 | 40-50 | 0.18 | 0.37 | 0.34 | 0.21 | 0.11 |
|  | 10-20 | 0-10 | 0.41 | 0.36 | 0.34 | 0.27 | 0.58 |
|  | 10-20 | 10-20 | 0.45 | 0.28 | 0.23 | 0.28 | 0.47 |
|  | 10-20 | 20-30 | 0.66 | 0.40 | 0.34 | 0.22 | 0.45 |
|  | 10-20 | 30-40 | 0.23 | 0.29 | 0.22 | 0.65 | 0.40 |
|  | 10-20 | 40-50 | 0.09 | 0.10 | 0.20 | 0.31 | 0.46 |
|  | 20-30 | 0-10 | 0.15 | 0.31 | 0.33 | 0.64 | 0.38 |
|  | 20-30 | 10-20 | 2.33 | 0.44 | 0.41 | 0.18 | 0.30 |
|  | 20-30 | 20-30 | 7.02 | 1.01 | 0.85 | 0.75 | 0.54 |
|  | 20-30 | 30-40 | 0.59 | 0.40 | 0.42 | 0.12 | 0.22 |
|  | 20-30 | 40-50 | 0.50 | 0.40 | 0.27 | 0.27 | 0.15 |
|  | 30-40 | 0-10 | 0.09 | 0.53 | 0.28 | 0.11 | 0.13 |
|  | 30-40 | 10-20 | 0.94 | 0.70 | 0.41 | 0.28 | 0.26 |
|  | 30-40 | 20-30 | 3.06 | 0.74 | 0.42 | 0.51 | 0.18 |
|  | 30-40 | 30-40 | 0.75 | 0.27 | 0.34 | 0.56 | 0.23 |
|  | 30-40 | 40-50 | 0.46 | 0.58 | 0.28 | 0.24 | 0.11 |
|  | 40-50 | 0-10 | 0.80 | 0.36 | 0.34 | 0.20 | 0.11 |
|  | 40-50 | 10-20 | 0.55 | 0.31 | 0.23 | 0.24 | 0.10 |
|  | 40-50 | 20-30 | 0.50 | 0.33 | 0.15 | 0.22 | 0.11 |
|  | 40-50 | 30-40 | 0.57 | 0.33 | 0.20 | 0.21 | 0.15 |
|  | 40-50 | 40-50 | 0.10 | 0.25 | 0.25 | 0.20 | 0.13 |
| RT | 0-10 | 0-10 | 0.08 | 0.07 | 0.12 | 0.13 | 0.15 |
|  | 0-10 | 10-20 | 0.20 | 0.67 | 0.21 | 0.20 | 0.11 |
|  | 0-10 | 20-30 | 0.12 | 0.56 | 0.15 | 0.34 | 0.43 |
|  | 0-10 | 30-40 | 0.53 | 0.24 | 0.17 | 0.15 | 0.28 |
|  | 0-10 | 40-50 | 0.20 | 0.54 | 0.30 | 0.07 | 0.08 |
|  | 10-20 | 0-10 | 0.46 | 0.52 | 0.30 | 0.11 | 0.42 |
|  | 10-20 | 10-20 | 0.51 | 0.41 | 0.33 | 0.24 | 0.34 |
|  | 10-20 | 20-30 | 0.75 | 0.59 | 0.31 | 0.19 | 0.33 |
|  | 10-20 | 30-40 | 0.26 | 0.43 | 0.33 | 0.56 | 0.29 |
|  | 10-20 | 40-50 | 0.10 | 0.15 | 0.17 | 0.26 | 0.33 |
|  | 20-30 | 0-10 | 0.17 | 0.45 | 0.28 | 0.55 | 0.27 |
|  | 20-30 | 10-20 | 2.64 | 0.65 | 0.30 | 0.15 | 0.22 |
|  | 20-30 | 20-30 | 7.94 | 1.48 | 0.67 | 0.45 | 0.38 |
|  | 20-30 | 30-40 | 0.67 | 0.58 | 0.32 | 0.20 | 0.16 |
|  | 20-30 | 40-50 | 0.56 | 0.59 | 0.23 | 0.23 | 0.11 |
|  | 30-40 | 0-10 | 0.11 | 0.77 | 0.24 | 0.21 | 0.09 |
|  | 30-40 | 10-20 | 1.06 | 1.03 | 0.34 | 0.24 | 0.18 |
|  | 30-40 | 20-30 | 3.46 | 1.08 | 0.35 | 0.44 | 0.13 |
|  | 30-40 | 30-40 | 0.84 | 0.39 | 0.35 | 0.48 | 0.17 |
|  | 30-40 | 40-50 | 0.52 | 0.85 | 0.24 | 0.21 | 0.06 |
|  | 40-50 | 0-10 | 0.90 | 0.53 | 0.34 | 0.10 | 0.06 |
|  | 40-50 | 10-20 | 0.62 | 0.45 | 0.35 | 0.21 | 0.10 |
|  | 40-50 | 20-30 | 0.56 | 0.48 | 0.13 | 0.19 | 0.08 |
|  | 40-50 | 30-40 | 0.65 | 0.48 | 0.17 | 0.18 | 0.10 |
|  | 40-50 | 40-50 | 0.11 | 0.36 | 0.21 | 0.15 | 0.10 |
| NT | 0-10 | 0-10 | 0.10 | 0.18 | 0.40 | 0.16 | 0.11 |
|  | 0-10 | 10-20 | 0.26 | 0.99 | 0.58 | 0.48 | 0.35 |
|  | 0-10 | 20-30 | 0.24 | 0.32 | 0.26 | 0.28 | 0.16 |
|  | 0-10 | 30-40 | 0.25 | 0.20 | 0.11 | 0.11 | 0.16 |
|  | 0-10 | 40-50 | 0.19 | 0.28 | 0.25 | 0.14 | 0.19 |
|  | 10-20 | 0-10 | 0.15 | 0.48 | 0.35 | 0.17 | 0.39 |
|  | 10-20 | 10-20 | 0.35 | 0.13 | 0.28 | 0.33 | 0.24 |
|  | 10-20 | 20-30 | 0.57 | 0.47 | 0.18 | 0.29 | 0.29 |
|  | 10-20 | 30-40 | 0.48 | 0.23 | 0.22 | 0.40 | 0.15 |
|  | 10-20 | 40-50 | 0.21 | 0.41 | 0.48 | 0.21 | 0.27 |
|  | 20-30 | 0-10 | 0.67 | 0.18 | 0.19 | 0.51 | 0.38 |
|  | 20-30 | 10-20 | 2.72 | 0.46 | 0.24 | 0.18 | 0.12 |
|  | 20-30 | 20-30 | 8.46 | 1.71 | 0.47 | 0.35 | 0.32 |
|  | 20-30 | 30-40 | 1.52 | 0.62 | 0.28 | 0.03 | 0.13 |
|  | 20-30 | 40-50 | 0.17 | 0.78 | 0.23 | 0.22 | 0.29 |
|  | 30-40 | 0-10 | 0.26 | 0.14 | 0.23 | 0.14 | 0.25 |
|  | 30-40 | 10-20 | 1.19 | 0.61 | 0.30 | 0.38 | 0.17 |
|  | 30-40 | 20-30 | 1.41 | 0.68 | 0.39 | 0.48 | 0.10 |
|  | 30-40 | 30-40 | 1.34 | 0.20 | 0.20 | 0.49 | 0.17 |
|  | 30-40 | 40-50 | 0.57 | 0.39 | 0.25 | 0.17 | 0.10 |
|  | 40-50 | 0-10 | 0.75 | 0.30 | 0.22 | 0.05 | 0.06 |
|  | 40-50 | 10-20 | 0.56 | 0.17 | 0.19 | 0.07 | 0.05 |
|  | 40-50 | 20-30 | 0.22 | 0.16 | 0.18 | 0.14 | 0.19 |
|  | 40-50 | 30-40 | 0.52 | 0.13 | 0.05 | 0.08 | 0.03 |
|  | 40-50 | 40-50 | 0.17 | 0.51 | 0.20 | 0.10 | 0.04 |

**Table F.** Coordination distribution of soil moisture, total nitrogen and root system of maize in 0–50 cm depths at VT under three tillage managements in 2011

| Soil depth (cm) | Y direction (cm) | Soil moisture content (g/g) | | | Total nitrogen concentration (mg/g) | | | Root length density (cm/cm^3^) | | |
| --- | --- | --- | --- | --- | --- | --- | --- | --- | --- | --- |
|  |  | SS | RT | NT | SS | RT | NT | SS | RT | NT |
| 0-10 | 0-10 | 0.11 | 0.10 | 0.12 | 1.10 | 1.12 | 1.11 | 0.15 | 0.17 | 0.67 |
| 0-10 | 10-20 | 0.09 | 0.08 | 0.11 | 1.05 | 0.93 | 1.15 | 2.33 | 2.64 | 2.72 |
| 0-10 | 20-30 | 0.11 | 0.08 | 0.11 | 1.04 | 0.90 | 1.11 | 7.02 | 7.94 | 8.46 |
| 0-10 | 30-40 | 0.10 | 0.08 | 0.13 | 1.01 | 0.93 | 1.25 | 0.59 | 0.67 | 1.52 |
| 0-10 | 40-50 | 0.10 | 0.10 | 0.13 | 1.09 | 1.11 | 1.11 | 0.50 | 0.56 | 0.17 |
| 10-20 | 0-10 | 0.13 | 0.12 | 0.12 | 0.97 | 1.12 | 0.99 | 0.31 | 0.45 | 0.18 |
| 10-20 | 10-20 | 0.13 | 0.10 | 0.12 | 1.33 | 0.94 | 1.08 | 0.44 | 0.65 | 0.46 |
| 10-20 | 20-30 | 0.11 | 0.10 | 0.10 | 1.04 | 0.99 | 0.92 | 1.01 | 1.48 | 1.71 |
| 10-20 | 30-40 | 0.11 | 0.10 | 0.11 | 0.83 | 0.87 | 1.04 | 0.40 | 0.58 | 0.62 |
| 10-20 | 40-50 | 0.12 | 0.12 | 0.13 | 0.80 | 0.85 | 1.00 | 0.40 | 0.59 | 0.78 |
| 20-30 | 0-10 | 0.11 | 0.11 | 0.10 | 0.88 | 0.84 | 0.81 | 0.33 | 0.28 | 0.19 |
| 20-30 | 10-20 | 0.12 | 0.11 | 0.11 | 1.07 | 0.75 | 0.68 | 0.41 | 0.30 | 0.24 |
| 20-30 | 20-30 | 0.13 | 0.11 | 0.11 | 0.90 | 0.74 | 0.69 | 0.85 | 0.67 | 0.47 |
| 20-30 | 30-40 | 0.11 | 0.11 | 0.10 | 0.86 | 0.65 | 0.64 | 0.42 | 0.32 | 0.28 |
| 20-30 | 40-50 | 0.14 | 0.12 | 0.11 | 0.82 | 0.63 | 0.62 | 0.27 | 0.23 | 0.23 |
| 30-40 | 0-10 | 0.14 | 0.10 | 0.11 | 0.80 | 0.59 | 0.59 | 0.64 | 0.55 | 0.51 |
| 30-40 | 10-20 | 0.15 | 0.10 | 0.12 | 1.00 | 0.59 | 0.61 | 0.18 | 0.15 | 0.18 |
| 30-40 | 20-30 | 0.14 | 0.10 | 0.12 | 0.96 | 0.55 | 0.66 | 0.75 | 0.45 | 0.35 |
| 30-40 | 30-40 | 0.14 | 0.13 | 0.11 | 0.91 | 0.68 | 0.60 | 0.12 | 0.20 | 0.03 |
| 30-40 | 40-50 | 0.14 | 0.13 | 0.12 | 0.89 | 0.60 | 0.59 | 0.27 | 0.23 | 0.22 |
| 40-50 | 0-10 | 0.15 | 0.14 | 0.14 | 0.68 | 0.62 | 0.60 | 0.38 | 0.27 | 0.38 |
| 40-50 | 10-20 | 0.14 | 0.16 | 0.15 | 0.68 | 0.73 | 0.67 | 0.30 | 0.22 | 0.12 |
| 40-50 | 20-30 | 0.13 | 0.14 | 0.15 | 0.72 | 0.67 | 0.72 | 0.54 | 0.38 | 0.32 |
| 40-50 | 30-40 | 0.13 | 0.13 | 0.15 | 0.68 | 0.59 | 0.65 | 0.22 | 0.16 | 0.13 |
| 40-50 | 40-50 | 0.14 | 0.13 | 0.15 | 1.10 | 1.12 | 1.11 | 0.15 | 0.11 | 0.29 |
